# Low Vitamin-D Levels Combined with *PKP3-SIGIRR-TMEM16J* Host Variants Is Associated with Tuberculosis and Death in HIV-Infected and -Exposed Infants

**DOI:** 10.1371/journal.pone.0148649

**Published:** 2016-02-12

**Authors:** Amita Gupta, Grace Montepiedra, Akshay Gupte, Bret Zeldow, Jennifer Jubulis, Barbara Detrick, Avy Violari, Shabir Madhi, Raziya Bobat, Mark Cotton, Charles Mitchell, Stephen Spector, IMPAACT NWCS113 and P1041 Study Team

**Affiliations:** 1 Johns Hopkins University School of Medicine, Baltimore, MD, United States of America; 2 Johns Hopkins Bloomberg School of Public Health, Baltimore, MD, United States of America; 3 Harvard School of Public Health, Boston, MA, United States of America; 4 Perinatal HIV Research Unit, Faculty of Health Sciences, University of the Witwatersrand, Johannesburg, South Africa; 5 Medical Research Council: Respiratory and Meningeal Pathogens Research Unit and Department of Science and Technology/National Research Foundation: Vaccine Preventable Diseases, University of the Witwatersrand, Johannesburg, South Africa; 6 Department of Pediatrics, University of KwaZulu-Natal, Durban, South Africa; 7 Children’s Infectious Diseases Clinical Research Unit, Faculty of Medicine and Health Sciences, Stellenbosch University, Stellenbosch, South Africa; 8 University of Miami, Miami, FL, United States of America; 9 University of California San Diego, La Jolla, CA, United States of America, and Rady Children’s Hospital, San Diego, CA, United States of America; Faculty of Medicine, AUSTRALIA

## Abstract

**Background:**

This study examined the associations of 25-hydroxyvitamin D and specific host genetic variants that affect vitamin D levels or its effects on immune function, with the risk of TB or mortality in children.

**Methods:**

A case-cohort sample of 466 South African infants enrolled in P1041 trial (NCT00080119) underwent 25-hydroxyvitamin D testing by chemiluminescent immunoassay. Single nucleotide polymorphisms (SNPs) that alter the effect of vitamin D [e.g. vitamin D receptor (VDR)], vitamin D levels [e.g. vitamin D binding protein (VDBP)], or toll like receptor (TLR) expression (*SIGIRR* including adjacent genes *PKP3* and *TMEM16J*) were identified by real-time PCR. Outcomes were time to TB, and to the composite of TB or death by 192 weeks of follow-up. Effect modification between vitamin D status and SNPs for outcomes was assessed.

**Findings:**

Median age at 25-hydroxyvitamin D determination was 8 months; 11% were breastfed, 51% were HIV-infected and 26% had low 25-hydroxyvitamin D (<32ng/mL). By 192 weeks, 138 incident TB cases (43 definite/probable, and 95 possible) and 26 deaths occurred. Adjusting for HIV status and potential confounders, low 25-hydroxyvitamin D was associated with any TB (adjusted hazard ratio [aHR] 1.76, 95% CI 1.01–3.05; p = 0.046) and any TB or death (aHR 1.76, 95% CI 1.03–3.00; p = 0.038). Children with low 25-hydroxyvitamin D and *TMEM 16J* rs7111432-AA or *PKP3* rs10902158-GG were at increased risk for probable/definite TB or death (aHR 8.12 and 4.83, p<0.05) and any TB or death (aHR 4.78 and 3.26, p<0.005) respectively; SNPs in VDBP, VDR, and vitamin D precursor or hydroxylation genes were not. There was significant interaction between low 25-hydroxyvitamin D and, *TMEM 16J* rs7111432-AA (p = 0.04) and *PKP3* rs10902158-GG (p = 0.02) SNPs.

**Conclusions:**

Two novel SNPs, thought to be associated with innate immunity, in combination with low vitamin D levels were identified as increasing a young child’s risk of developing TB disease or death. Identifying high-risk children and providing targeted interventions such as vitamin D supplementation may be beneficial.

**Trial Registration:**

ClinicalTrials.gov NCT00080119

## Introduction

Annually, more than 7.6 million children under 15 years of age become infected with *Mycobacterium tuberculosis* (*Mtb*), 650,000 develop tuberculosis disease (TB) and over 74,000 die [1–3]. A substantial number of childhood TB cases and deaths occur in HIV and TB endemic countries among HIV-infected and -exposed children under 5 years who are at high risk for developing severe forms of disseminated TB [4]. Prompt and effective diagnosis of TB in children using currently available diagnostic methods can be challenging [5]. Furthermore, treating severe forms of TB in children is resource intensive and is often associated with considerable morbidity and mortality. Thus, strategies aimed at identifying children who are at high risk of developing TB disease are critical.

In adults, several host factors appear to increase the risk of developing TB or death. For example, low levels of 25-hydroxyvitamin D (i.e. vitamin D insufficiency and deficiency), are associated with increased risk of TB infection, TB disease and severity of disease [6–8]. Although the precise mechanisms through which adequate vitamin D protects against TB are unknown, it is thought to enhance innate immunity by regulating monocyte activation, T-cell suppression and cytokine synthesis [9]. Furthermore, several host genetic single nucleotide polymorphisms (SNPs) impacting the vitamin D binding protein (VDBP) as well as the vitamin D receptor (VDR) have also been linked to increased risk of TB, though with variable quality of evidence [10–14]. More recently, a significant association between SNPs in the single immunoglobulin interleukin 1 (IL-1) receptor (*SIGIRR*, also known as Toll IL-1 receptor 8 [Toll/IL-1R-8, TIR8] region including adjacent SNPs in plakophilin 3 (*PKP3*) and transmembrane 16J (*TMEM16J*) were associated with susceptibility to TB in Vietnam [15]. This gene region is associated with innate immune pathways and adaptive immune responses, and acts as a negative regulator of Toll-like-receptor (TLR) signaling which is important in *Mtb* pathogenesis [16, 17]. Interestingly, the antimicrobial activity associated with TLR signaling in macrophages was shown to be dependent on the vitamin D pathway [18, 19] and the association between low levels of 25-hydroxyvitamin D and TB can be modified by host genotype [8, 20, 21].

Whether the associations between low 25-hydroxyvitamin D, host VDR and other SNPs associated with TB risk in adults are important to pediatric populations, especially those in high TB and HIV burden settings, have not been examined. Young children are more likely to be dependent on innate immunity for control of infections prior to the development of mature adaptive immunity and are at high risk for developing TB and dying [4].

Thus in the current study, we examined the associations between low 25-hydroxyvitamin D and host genetic SNPs, including those in the *PKP3-SIGIRR-TMEM16J* region that are associated with an increased risk of TB [15], with TB and all-cause mortality among HIV-infected and –exposed infants in South Africa. We identify a novel interaction between serum vitamin D levels and genetic variants within the *PKP3-SIGIRR-TMEM16J* region, which is strongly associated with TB and death among young children.

## Methods

### Study design and population

This study had two objectives. First was to measure the prevalence and risk factors of low 25-hydroxyvitamin D in the first year of life. Second was to evaluate the associations between 25-hydroxyvitamin D, specific host SNPs including those in the VDR and VDBP genes as well as those altering innate immunity in the *PKP3-SIGIRR-TMEM16J* gene region and, probable or definite TB in HIV-infected and -exposed infants in a TB-HIV endemic region. Additional objectives were to evaluate the association between these SNPs and the risk of any TB (possible, probable, definite TB) as well as composite any TB or death (the primary endpoint in P1041). All TB outcomes were assessed up to 192 weeks of follow up. We used a case-cohort design to select 466 infants enrolled in the IMPAACT P1041 trial (Clinical Trials.gov NCT00080119) in South Africa; the details of this trial are described elsewhere [22]. Briefly, P1041 was a Phase II/III, randomized, double-blind, placebo-controlled clinical trial to evaluate the efficacy of isoniazid prophylaxis on TB disease and latent *Mtb* infection free survival in HIV-infected and HIV-exposed, but uninfected infants up to 192 weeks of follow up. The vast majority of the 1351 infants enrolled between 3 and 4 months of age were from three sites in South Africa (Johannesburg, Cape Town, and Durban); the one site in Botswana (Gaborone) contributed <0.5% of participants and was not included in our study. All infants were BCG vaccinated and had no previous known TB exposure. The DSMB recommend study closure after an interim analysis showed futility of the isoniazid prophylaxis in both cohorts.

**Fig 1:**
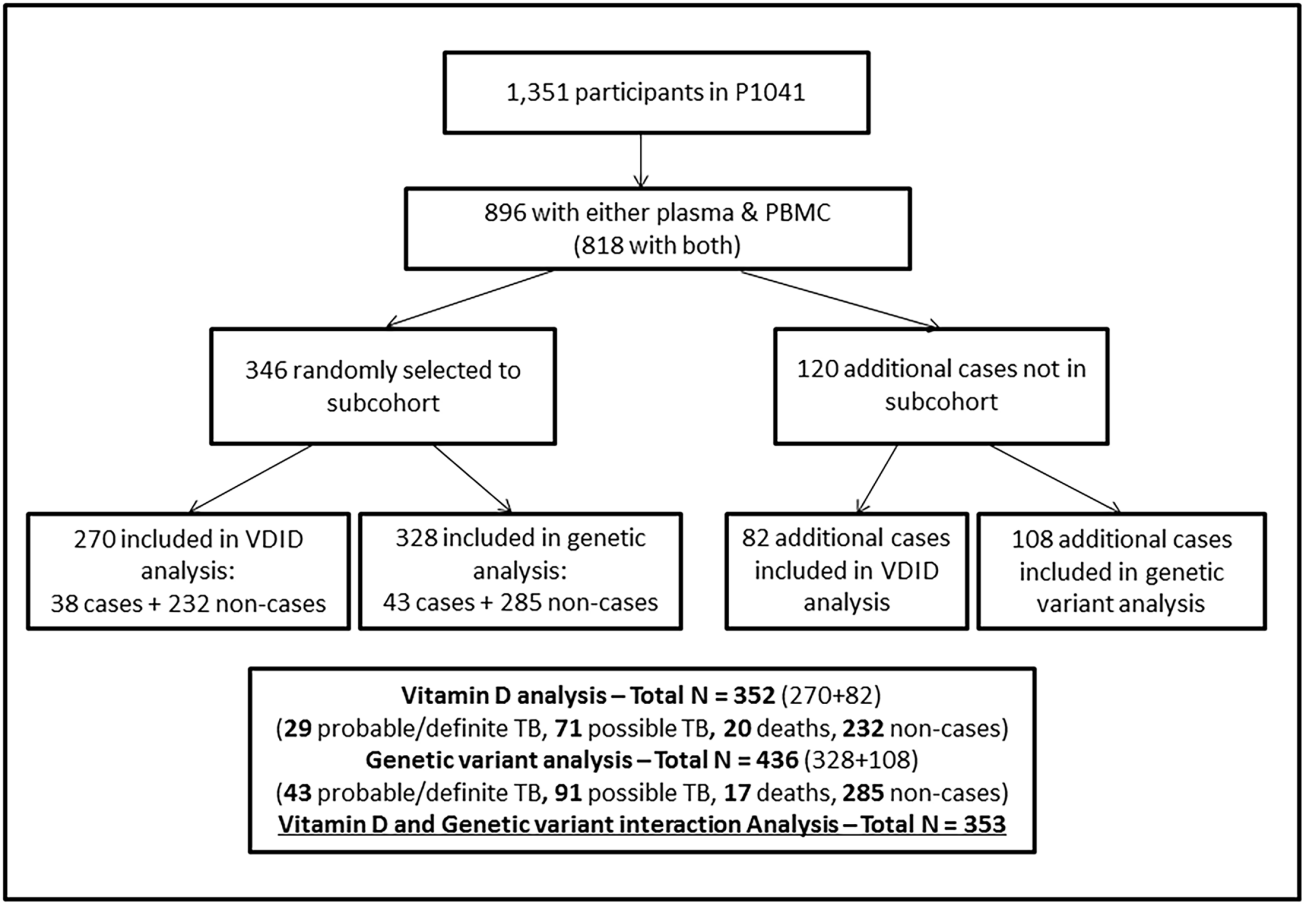
Consort diagram for case-cohort study.

We used a case-cohort design, which allows for both prevalence estimation of the exposures of interest and multiple outcome assessments without having to test the entire P1041 study population [23, 24]. Of 1,351 P1041 trial infants, 896 were eligible as they had either archived serum/plasma (at least 0.125 ml) or peripheral blood mononuclear cells (PBMCs) available; 818 had both. Samples were obtained from two groups: 1) subcohort group: a randomly selected group of 346 eligible infants with both serum/plasma and PBMC samples; 2) case group: 120 participants not included in the subcohort who experienced the outcomes of interest (TB or death). The subcohort group included children with TB. As is the convention with case-cohort studies, the subcohort was used for prevalence estimation and determination of risk factors for low 25-hydroxyvitamin D. The case group was only included in the assessment of association between 25-hydroxyvitamin D, host variants and outcomes of interest. For these analyses, the case group was combined with the subcohort group to form the case-cohort sample according to case-cohort design principles (352 for vitamin D analyses and 436 for host SNPs analyses). (Fig 1)

The analysis reported in this manuscript was conceived after completion of the P1041 trial. Study participants were enrolled between December 2004 and June 2008, and laboratory analyses on archived samples were performed in 2013. Written informed consent was obtained from parents or legal guardians on behalf of the children enrolled in our study. Our study was approved by the Institutional Review Boards and Ethics Committees at Johns Hopkins University, University of California San Diego, Stellenbosch University, University of Witwatersrand, Soweto, South Africa and the University of KwaZulu Natal, Durban, South Africa.

### Laboratory methods

#### Determination of 25-hydroxyvitamin D concentrations

Total 25-hydroxyvitamin D concentrations were assessed on archived serum from study entry or a study visit closest to entry into P1041 using the Diasorin^**®**^ chemiluminescent immunoassay. Testing was performed at Johns Hopkins University, an externally quality-assured laboratory. We defined vitamin D sufficient as values ≥32 ng/mL, 20 to 31 ng/mL as insufficient, and below 20 ng/mL as deficient [9, 25–27].

#### Genotyping

Using archived PBMCs, we examined SNPs associated with low 25-hydroxyvitamin D located within genes that encode VDBP [GC(rs2282679), rs7041, rs4588], 7-dehydrocholesterol reductase involved in synthesis of a vitamin D precursor molecule [DHCR7(rs12785878)] and a hepatic enzyme within the P450 subfamily involved in 25-hydroxylation of vitamin D [CYP2R1(rs10741657)]. Four VDR SNPs [*Fok-l* (rs10736810), *Bsm-l* (rs1544410), *Apa-1* (rs7975232) and *Taq-1* (rs731236)] and three SNPs in the *PKP3-SIGIRR-TMEM16J* gene region associated with TB susceptibility [*PKP3* (rs10902158-A/G), *PKP3* (rs7105848-T/C), and *TMEM16J* (rs7111432-G/A)][15] were also assessed by real-time PCR assays at the University of California, San Diego IMPAACT Network Specialty Laboratory.

### Statistical methods

#### Outcome definitions

Low vitamin D was defined as <32 ng/mL. TB was classified as definite, probable or possible based on the 2004 South African National TB Control Program Guidelines [28]. The diagnostic algorithm is described elsewhere [22]. A definite case was *Mtb* cultured from any site. A probable included at least two clinical criteria in the algorithm plus either positive auramine staining of an induced-sputum or gastric-washing smear or suggestive histologic findings (e.g. caseating granuloma); or positive auramine staining of a gastric washing or induced-sputum smear and a chest radiograph suggestive of TB. A possible case was a clinical algorithm score ≥6 and a radiograph suggestive of TB, or a positive tuberculin skin test (induration ≥5 mm in horizontal diameter) and a chest radiograph suggestive of TB [22]. Outcome of death included all-cause mortality and was not restricted to deaths due to TB.

#### Statistical analysis

Comparisons in baseline characteristics between infants selected for the subcohort and those not selected among those eligible were assessed using Chi-Square, Fisher’s exact, and Wilcoxon tests, as appropriate. The prevalence of low 25-hydroxyvitamin D and SNPs was calculated among the subcohort and 95% confidence intervals were constructed using Clopper-Pearson exact methodology.[29]

Baseline correlates of low 25-hydroxyvitamin D were assessed using Chi-Square tests. Cox Proportional Hazards regression models were built to determine the association between low 25-hydroxyvitamin D and outcomes of interest. We also explored Vitamin D level as a continuous variable in models. Formal tests were performed to check the assumption of proportionality of hazards, and no violations were detected. Models were adjusted for the case-cohort design by using Barlow, Prentice, and Self-Prentice weighting schemes [23, 24]. All models included low 25-hydroxyvitamin D as the main exposure of interest, adjusting for HIV status, site, season, sex, WHO weight z-score at baseline, whether or not the child was breastfed, type of housing, the mother having a previous TB diagnosis, age in months at plasma draw date and P1041 randomized treatment group of INH or placebo. Models with probable or definite TB as the outcome of interest did not adjust for whether or not the child was breastfed due to model stability issues. Association between low 25-hydroxyvitamin D and SNPs among the subcohort was assessed using Chi-Square tests. Associations between SNPs and the study outcomes described above were assessed using Cox regression models, adjusting for HIV and vitamin D status, and included models that additionally adjusted for the other covariates described above (except for season and age at plasma draw date). Interactions between SNPs and 25-hydroxyvitamin D status were examined and combinations of 25 hydroxyvitamin D status and SNPs were compared. For all Cox Proportional Hazards regression models involving 25-hydroxyvitamin D status, the plasma draw date (date of earliest available sufficient plasma draw) was the time at which participants were first considered at-risk. However, for cases when polymorphisms were considered without vitamin D status included in the model, participants were considered at-risk beginning at study entry for P1041. We applied the methodology of Mark and Katki [30] to construct Kaplan-Meier (K-M) curves using the full case-cohort. This method is equivalent to using Self-Prentice weights when the case-cohort sample size is large, such as in this study. Risk differences were computed as the differences in Kaplan-Meier survival estimates at end of follow-up period for this analysis, and were not adjusted for covariates.

The sample size for the sub-cohort corresponds to a sampling fraction of about 30% of the total cohort size of eligible subjects, since this sampling fraction provides nearly the same power to detect significant hazard ratios for vitamin D deficiency/insufficiency (assuming vitamin D deficiency/insufficiency prevalence of 30%) as when the full cohort of eligible subjects were used in the analysis. Similarly, this sampling fraction provides nearly the same power to detect significant hazard ratios for genetic variants (assuming genotype prevalence in the range 20%-50%) as when the full cohort were used in the analysis.

Tests resulting in p-value < 0.05 are reported as significant and 0.05 < p-value < 0.10 are reported as marginally significant. Adjustments for multiple testing of exposures of interest (vitamin D level, genetic variants, and interactions) within each outcome of interest, using Benjamini-Hochberg approach at false discovery rates of FDR = 0.05 and 0.10, are also reported.

Analyses were performed in SAS, version 9.2. K-M curves were constructed using the NestedCohort package (Version 1.1–2) in R, Version 2.15.1.

## Results

### Characteristics of study participants

A total of 466 infants comprised the case-cohort sample (346 in the randomly selected subcohort and 120 in the case group meeting outcomes of interest but not in the subcohort). There were 134 TB cases (43 definite or probable and 91 possible cases) and 26 deaths. Of TB cases, 131 were pulmonary and 3 were extrapulmonary. Cause of death included gastroenteritis (n = 7), pneumonia (n = 6), sepsis (n = 3), other (n = 4) and unknown (n = 6). Among the 215 HIV-infected infants in the full case-cohort, 27 (13%) were never on ARVs prior to or during the study, 61 (28%) were on ARVs prior to entering the study and 127 (59%) initiated ARVs during the study, with a median time to ART initiation (from enrollment) of 27 days in the latter group. 169 (90%) infants received either Stavudine, Lamivudine and Lopinavir/ritonavir or Lamivudine, Zidovudine and Lopinavir/ritonavir combination therapy. None of the infants received Efavirenz or Tenofovir based regimes. In the subcohort, 167 (48%) infants were males, 148 (43%) were HIV-infected, 58 (17%) had low birth weight and 39 (11%) were ever breast-fed (Table 1). Infants included in the subcohort were similar to those not included but eligible, with the exception of site of enrollment (data not shown). There were significant differences between children eligible (with relevant samples for the data analysis) and those ineligible with respect year of enrollment into P1041 (p-value<0.001), HIV status (p-value<0.001), site of enrollment (p-value<0.001), weight z-score (p-value<0.001), whether there were persons >55 years old in the household or not (p-value = 0.002), housing type (p-value<0.001) and water access (p-value<0.001), and there was marginally significant difference with respect to whether the infant was breastfeeding or not at study entry (p-value = 0.09). (S1 Table)

**Table 1:** Baseline characteristics of full case-cohort (N = 466).

Characteristic	Full Case-cohort			
	Random sub-cohort			Non-subcohort cases (N = 120**)
	Infants chosen for subcohort (N = 346*)	Case of subcohort?		
		No (N = 302)	Yes (N = 44)	
**Infant**				
*Site*				
Johannesburg	271(78%)	233(77%)	38(86%)	90(75%)
Cape Town	49(14%)	47(16%)	2(5%)	20(17%)
Durban	26(8%)	22(7%)	4(9%)	10(8%)
*Male Sex*	167(48%)	143(47%)	24(55%)	51(43%)
*Mean (sd) age in months*	3.26(0.30)	3.26(0.30)	3.30(0.29)	3.28(0.29)
*Range of age in months*	2.99–3.94	2.99–3.94	2.99–3.91	2.99–3.94
*HIV-infected*	148(43%)	121(40%)	27(61%)	67(56%)
*INH Treatment Group*	159(46%)	141(47%)	18(41%)	56(47%)
*Birth Weight<2500 gms*	58(17%)	50(17%)	8(18%)	19(16%)
*Mean (sd) birth weight*	2939(562)	2951(565)	2859(544)	2891(482)
*Range of birth weight*	600–4350	600–4350	1360–3800	1490–4440
*Mean (sd)weight z-score*	-0.77(1.43)	-0.73(1.39)	-1.10(1.61)	-1.21(1.47)
*Range of weight z-score*	-5.84–3.48	-5.84–3.48	-5.19–1.21	-5.41–1.94
*Ever breastfed*	39(11%)	35(12%)	4(9%)	7(6%)
**Maternal and Household**				
*Mother Ever had TB Diagnosis*	24(7%)	22(7%)	2(5%)	11(9%)
*Housing Type*				
Formal (brick) house	241(70%)	210(70%)	31(70%)	77(64%)
Informal shack	105(30%)	92(30%)	13(30%)	42(35%)
*Water access*				
Running water in home	124(36%)	108(36%)	16(36%)	39(33%)
Running water on plot	162(47%)	141(47%)	21(48%)	52(43%)
Communal tap	60(17%)	53(17%)	7(16%)	29(24%)

*Of the 346 samples, 333 had evaluable vitamin D or genetic polymorphism data.

** Only the 120 non-cohort cases with evaluable vitamin D or genetic polymorphism data are included. The sub-cohort (n = 346) was the random sample from the P1041 trial for whom adequate plasma and peripheral blood samples were available. The random sub-cohort had 44 evaluable cases (16 probable/confirmed, 27 possible and 1 death). There were an additional 120 evaluable cases (27 probable/confirmed, 68 possible and 25 deaths) that contributed to a total evaluable case sample of 164.

### Prevalence and association of low 25-hydroxyvitamin D with TB and death

The mean age at blood draw for 25-hydroxyvitamin D assessments was 7.59 months. The prevalence of low 25-hydroxyvitamin D was 26% (95%CI 21–32), of which 2% were vitamin D deficient and 24% insufficient. (Table 2) Low 25-hydroxyvitamin D status was associated with younger age, being female, living in formal houses and birth weight ≥2500 grams (p = 0.048, 0.05, 0.004 and 0.02, respectively). (Table 3)

**Table 2:** Prevalence estimates of Vitamin D and host SNPs of interest in sub-cohort (N = 346***).

Characteristic	n(%**)
**25 hydroxyvitamin D status**	
Low* (<32 ng/mL)	70(26.0%)
Deficient (<20 ng/mL)	5(1.9%)
Insufficient (20–31 ng/mL)	65(24.1%)
Sufficient (> = 32 ng/mL)	200(74.1%)
Missing	76(NA)
**TMEM16J Genotype (rs7111432)**	
AA	83(25.3%)
AG	180(54.9%)
GG	65(19.8%)
**PKP3 Genotype (rs10902158)**	
AA	32(9.8%)
AG	153(46.6%)
GG	143(43.6%)
**PKP3 Genotype (rs7105848)**	
CC	31(9%)
CT	140(43%)
TT	157(48%)
**CYP2R1 Genotype (rs10741657)**	
AA	13(4.0%)
AG	100(30.5%)
GG	215(65.6%)
**DHCR7 Genotype (rs12785878)**	
GG	218(66.5%)
GT	98(29.9%)
TT	12(3.7%)
**VDBP Genotype (rs2282679)**	
AA	287(87.5%)
AC	39(11.9%)
CC	2(0.6%)
**VDBP Genotype (rs4588)**	
AC	36(11.0%)
CC	292(89.0%)
**VDBP Genotype (rs7041)**	
GG	3(0.9%)
GT	39(11.9%)
TT	286(87.2%)
**VDR *bsm* Genotype (rs1544410)**	
AA	14(4.3%)
AG	115(35.1%)
GG	199(60.7%)
**VDR *fok-1* Genotype (rs10736810)**	
CC	227(69.2%)
CT	91(27.7%)
TT	10(3.1%)

*Vitamin D Insufficient or Deficient.

**Observed prevalence (excluded missing values in denominator).

***Missing genotype data for 18 subjects from sub-cohort of N = 346.

**Table 3:** Risk factors for low Vitamin D (25 hydroxyvitamin D) concentrations among the sub-cohort participants.

Characteristic	Total (N = 270)	Without low Vit D (N = 200)	With low Vit D (N = 70)	P-Value
*Site*				0.41**
Johannesburg	233	170(73%)	63(27%)	
Cape Town	12	11(92%)	1(8%)	
Durban	25	19(76%)	6(24%)	
***Infant characteristics***				
Mean *age at draw date*, *months* (s.d.)	7.59(1.93)	7.72(1.83)	7.21(2.18)	0.048*
*Sex*				0.05**
Male	128	102(80%)	26(20%)	
Female	142	98(69%)	44(31%)	
*HIV Status*				1.00**
HIV-infected	138	102(74%)	36(26%)	
HIV-uninfected	132	98(74%)	34(26%)	
*Birth Weight<2500 gms*				0.018**
Yes	49	43(88%)	6(12%)	
No	221	157(71%)	64(29%)	
*Mean weight z-score (WHO) (s*.*d*.*)*	-0.90(1.47)	-0.94(1.47)	-0.80(1.49)	0.45*
*Ever breastfed*?				1.00**
Yes	31	23(74%)	8(26%)	
No	239	177(74%)	62(26%)	
*Season of sample for Vitamin D*				
April-Sept	125	87(70%)	38(30%)	0.13**
Oct-March	145	113(78%)	32(22%)	
*Mother Ever had TB Diagnosis*				0.25**
Yes	16	14(88%)	2(13%)	
No	254	186(73%)	68(27%)	
***Household characteristics***				
*>3 people sleep in room*				0.68**
Yes	106	76(72%)	30(28%)	
No	163	123(75%)	40(25%)	
Not known	1	1(100%)	0(0%)	
*Any 55 year olds in house*?				0.44**
Yes	78	55(71%)	23(29%)	
No	192	145(76%)	47(24%)	
*Housing Type*				0.004**
Formal (brick) house	190	131(69%)	59(31%)	
Informal shack	80	69(86%)	11(14%)	
*Water access*				0.07**
Running water in home	94	67(71%)	27(29%)	
Running water on plot	134	96(72%)	38(28%)	
Communal tap	42	37(88%)	5(12%)	

*Wilcoxon test.

**Chi-square test.

**Fig 2:**
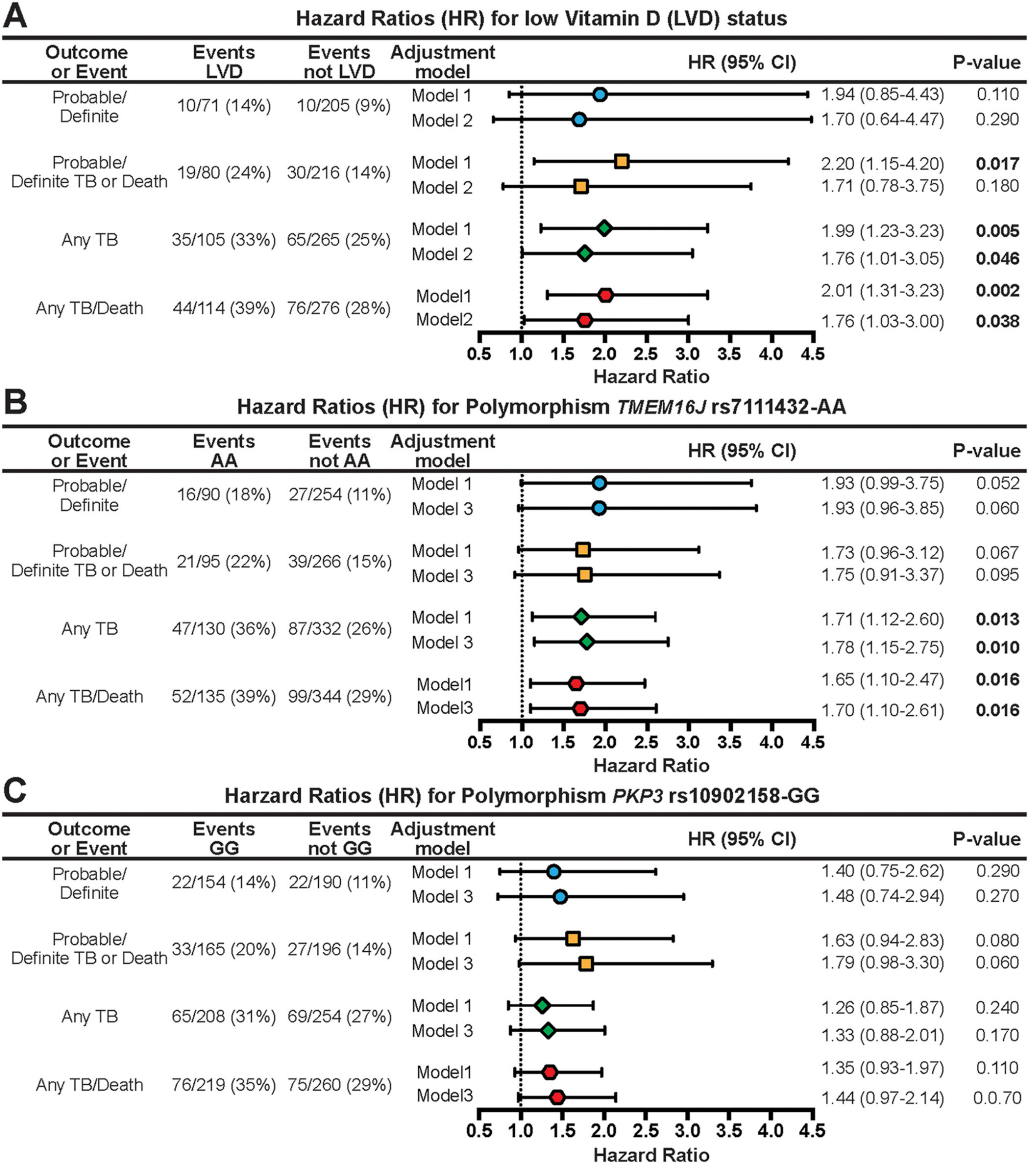
Cox Proportional Hazard Models for TB disease or death, looking at (A) low vitamin D (LVD) status as risk factor ignoring genetic variant, (B) *TMEM16J* rs7111432 variant as risk factor ignoring LVD status, and (C) *PKP3* rs10902158 variant as risk factor ignoring LVD status. Model 1 adjusts for HIV infection status. *Model 2 adjusts for HIV status*, *season*, *site*, *sex*, *weight z-score*, *type of house*, *mother prev TB diagnosis*, *age (in months) at plasma draw date*, *and INH/Placebo group*. *Model 3 adjusts for HIV status*, *site*, *sex*, *weight z-score*, *type of house*, *mother prev TB diagnosis*, *and INH/Placebo group*. Evts/LVD = no. of events/no. of subjects with low vitamin D (%). Evts/NotLVD = no. of events/no. of subjects without low vitamin D (%). Evts/AA = no. of events/no. of subjects with AA genotype (%). Evts/NotAA = no. of events/no. of subjects without AA genotype (%). Evts/GG = no. of events/no. of subjects with GG genotype (%). Evts/NotGG = no. of events/no. of subjects without GG genotype (%).

**Fig 3:**
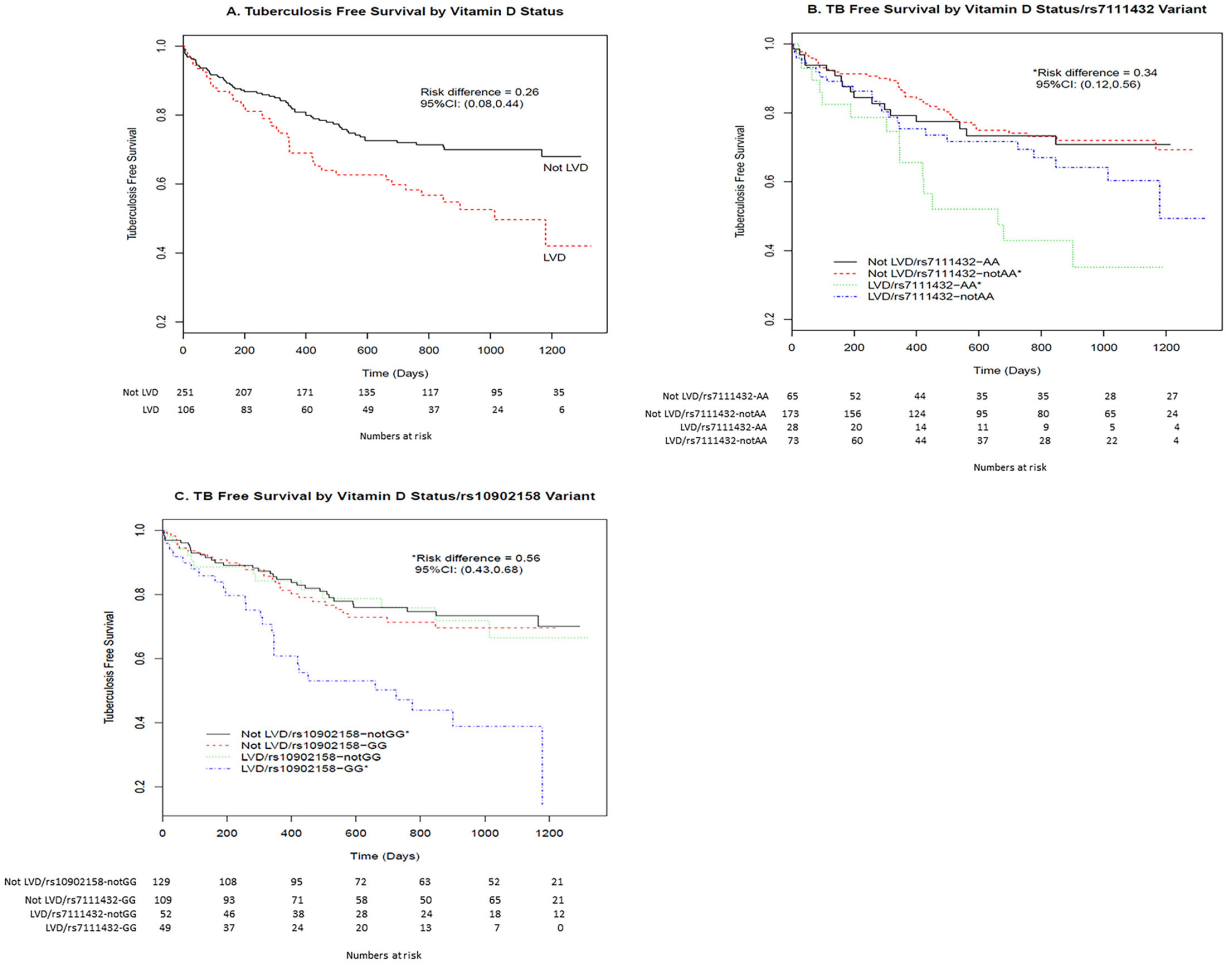
Kaplan-Meier Plots on Tuberculosis Free Survival (from all available data in case-cohort) by (A) Vitamin D status, (B) Vitamin D status / rs7111432 variant and (C) Vitamin D status / rs10902158 variant. Risk differences were computed as the differences in Kaplan-Meier survival estimates at end of follow-up period for this analysis, and were not adjusted for covariates.

In analyses only adjusting for HIV status, infants with low 25-hydroxyvitamin D had approximately a 2-fold greater risk of being diagnosed with probable or definite TB (aHR 1.70, 95%CI 0.64–4.47; p = 0.29, not significant), any TB (aHR 1.99, 95%CI 1.23–3.23; p = 0.005, significant), and any TB or death (aHR 2.01; 95%CI 1.31–3.23; p = 0.002, significant) as compared to their vitamin D sufficient counterparts.(Fig 2A) In multivariable analyses, adjusting for HIV, site, season, birth weight, breastfeeding, age at plasma draw, house, and INH/placebo group, low 25-hydroxyvitamin D remained significantly associated with any TB (aHR 1.76, 95%CI 1.01–3.05;p = 0.046) and any TB or death (aHR 1.76, 95%CI 1.03–3.00,p = 0.038).(Fig 2A) For the outcome of any TB or death, the survival (K-M) curve for infants with low vitamin D levels was significantly lower than those with normal vitamin D levels (unadjusted risk difference 0.26, 95%CI 0.08–0.44; Fig 3A).

We assessed vitamin D as a continuous variable in models on all outcomes of interest, ignoring the vitamin D polymorphisms, but found no significant result (data not shown).

### Prevalence and association of SNPs with vitamin D levels, TB and death

The prevalence of allelic frequencies of SNPs assessed is shown in Table 2. None of the SNPs evaluated were associated with an increased risk of low 25-hydroxyvitamin D (S2 Table). Additionally, no SNPs examined within the VDBP (rs2282679, rs4588, rs7041), VDR (rs10736810, rs1544410, rs7975232, rs731236) or those reportedly associated with vitamin D levels (rs12785878, rs10741657) significantly altered the risk for any TB or death (S3 Table). There were, however, marginally significant associations between rs7041 and probable/definite TB or death (aHR 3.25, 95%CI 0.95–11.1; p = 0.06; S3 Table).

In contrast to the findings relating to the vitamin D related SNPs, SNPs within the *PKP3-SIGIRR-TMEM16J* region significantly altered the risk for TB or death. In crude and adjusted Cox models, having the *TMEM16J* (rs7111432) AA-allele was associated with approximately a two-fold-increased risk of probable or definite TB (aHR 1.93, 95%CI 0.96–3.85; p = 0.06, marginally significant), any TB (aHR 1.75, 95%CI 0.91–3.37; p = 0.095, marginally significant) and any TB or death (aHR 1.70, 95%CI 1.10–2.61; p = 0.016, significant). (Fig 2B) Similarly, having the *PKP3* (rs10902158) GG-allele was marginally associated with an increased risk of any TB or death (aHR 1.44 95% CI 0.97–2.14; p = 0.07).(Fig 2C)

### Low 25-hydroxyvitamin D status with SNPs in the PKP3-SIGIRR-TMEM16J region genetic variants identify infants at high risk for TB and death

We next examined whether infants with low 25-hydroxyvitamin D who also had specific SNPs within the *PKP3-SIGIRR-TMEM16J* region would be at greater risk for TB or death. We hypothesized that there would be an increased risk for TB or death because vitamin D has an important role in innate immunity through autophagy induction [31, 32] and SNPs within the *PKP3-SIGIRR-TMEM16J* region impair the down-regulation of toll-like receptor IL-1R signaling, a pathway critical to host immune responses to *Mtb* [15]. Consistent with our hypothesis, there was a significant interaction between vitamin D status (low 25-hydroxyvitamin D or not) and *TMEM16J* rs7111432 variants (GG genotype or not) in the adjusted models for any TB (p = 0.04) and any TB or death (p = 0.04). There was also a significant interaction between vitamin D status and *PKP3* rs10902158 variants (AA genotype or not) in the adjusted models for any TB or death (p = 0.02).

**Fig 4:**
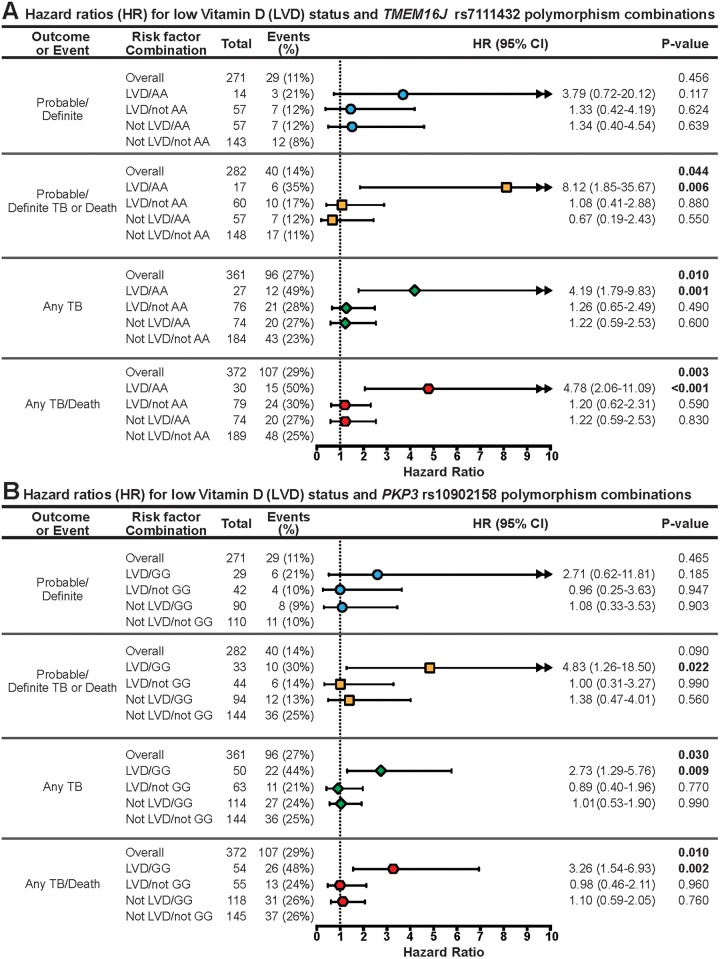
Cox Proportional Hazard Models for TB disease or death, looking at (A) combination of low Vitamin D (LVD) status and *TMEM 16J* rs7111432 variant [AA genotype or not] as risk factor, and (B) combination of LVD status and *PKP3* rs10902158 variant [GG genotype or not] as risk factor. A: Model adjusts for HIV status, season, site, sex, weight z-score, type of house, mother with previous TB diagnosis, age (in months) at plasma draw date, and INH/Placebo group. B: Model adjusts for HIV status, season, site, sex, weight z-score, type of house, mother with previous TB diagnosis, age (in months) at plasma draw date, and INH/Placebo group.

With combinations of vitamin D status and *TMEM 16J* rs7111432 variants as independent variables in adjusted Cox proportional hazard models, we found that infants with low 25-hydroxyvitamin D and rs7111432-AA genotype had more than a 6-fold increased risk of probable or definite TB (aHR = 6.54, 95%CI 1.07–39.83 p = 0.04, significant) and 8-fold increased risk of probable/definite TB or death (HR = 8.12 95% CI 1.85–35.67 p = 0.006, significant) compared to those without low vitamin D and without AA genotype. When including possible TB events, the risk remained more than 4-fold increased (any TB HR = 4.19 95% CI 1.79–9.83 p = 0.001, significant; and any TB or death HR = 4.78 95% CI 2.06–11.09 p = 0.0003, significant). (Fig 4A) Adjusting for multiple testing, and using a false discovery rate of FDR = 0.05, the comparison of risk of any TB or death between subjects with combination of LVD / rs7111432-AA genotype versus those with combination of not LVD / rs7111432-not AA genotype remained significant. For any TB or death, the K-M plot shows a much lower survival curve among infants with low vitamin D levels and with the AA genotype compared to the three more similar curves representing the other combinations (e.g., unadjusted risk difference versus infants with normal vitamin D level and with the AG/GG genotype is 0.34 with 95%CI 0.12–0.56; Fig 3B).

Similarly, models fitted with a combination of vitamin D status and *PKP3* rs10902158 variants as independent variables demonstrated that having low 25-hydroxyvitamin D and rs10902158-GG genotype had a significantly higher risk of probable/definite TB or death (aHR = 4.83, 95%CI 1.26–18.50; p = 0.02) as well as any TB (aHR 2.73, 95%CI 1.29–5.75; p = 0.009) or any TB or death (3.26, 95%CI 1.54–6.93; p = 0.002). (Fig 4B) Adjusting for multiple testing, and with an FDR = 0.10, the comparison of risk of any TB or death between subjects with combination of LVD / rs10902158-GG genotype versus those with combination of not LVD / rs10902158-not GG genotype remained significant. For the outcome of any TB or death, the K-M plots are shown in Fig 3C shows the much lower survival curve among infants with low vitamin D levels and with the rs10902158-GG genotype compared to the three more similar curves representing the other combinations (e.g., unadjusted risk difference versus infants with normal vitamin D level and with the AA/AG genotype is 0.56 with 95%CI 0.43–0.68).

## Discussion

This study has several key findings. First, 26% of young children residing in a high TB and HIV burden setting had low levels of 25-hydroxyvitamin D. Second, low 25-hydroxyvitamin D below 32 ng/mL was associated with a 1.7-fold increased risk for TB and death independent of host variants. Third, we demonstrate that genetic variants within the *PKP3-SIGIRR-TMEM16J* gene region (*PKP3*-rs10902158-A/G and *TMEM16J*- rs7111432 G/A) are significantly associated with a 40% to 70% increased risk for pediatric TB and death. Lastly, the most intriguing and relevant finding was the interaction between low 25-hydroxyvitamin D and *PKP3-SIGIRR-TMEM16J* host variants. To our knowledge, we demonstrate for the first time that young children with both low 25-hydroxyvitamin D and the rs7111432-AA have a 4- to 8-fold increased risk of TB or death. Similarly, those with both low 25-hydroxyvitamin D and rs10902158-GG genotypes have a 3- to 4-fold increased risk for developing TB or dying. It is likely that such effect modifying variants will have important implications for vitamin D supplementation strategies. Vitamin D supplementation may have greatest benefit in preventing TB in children with both low levels of 25-hydroxyvitamin D and *PKP3-SIGIRR-TMEM16J* host variants reported in our study.

We observed that 26% of infants were vitamin D insufficient or deficient. This prevalence is lower than reported elsewhere [33]. In agreement with the infant feeding policy for HIV exposed infants in South Africa at the time the study was conducted, a high proportion of the children in our study were fed vitamin D fortified formula. As would be expected, few infants were identified to be vitamin D deficient using the criterion of <20 ng/mL. For the purposes of immunologic health, we apriori used a value of ≥32 ng/mL 25-hydroxyvitamin D as sufficient. In support of using a higher value of vitamin D for optimal immunologic health, Campbell and Spector demonstrated that the activity of vitamin D3 against *Mtb* follows a dose response with higher concentrations providing greater killing of the organism [34].

Low levels of vitamin D has long been implicated in the susceptibility to TB [35]. In our study, children with low 25-hydroxyvitamin D had a 70% increased risk of developing any TB or death. Molecular mechanisms responsible for the activity of vitamin D against *Mtb* are the subject of considerable research [8]. For children who are infected with *Mtb* but do not develop disease, the infectious organisms can remain within the phagosomes of macrophages and may not reactivate for decades [9]. *Mtb* prevents the acidification of the phagosomes preventing the maturation to phagolysosomes and the degradation of the mycobacteria. The trafficking pathway whereby cytoplasmic constituents, including sub-cellular organelles, and microbial pathogens, such as *Mtb*, are engulfed by phagosomes (or autophagosomes) which fuse with lysosomes forming phagolysosomes (or autolysosomes) leading to bulk degradation is termed autophagy. Numerous studies have demonstrated that the induction of autophagy *in vitro* can kill *Mtb* [31, 32]. In this regard, the active form of vitamin D (1,25D3) can induce autophagy with the anti-mycobacterial activity dependent on the induction of autophagy and the expression of endogenous human cathelicidin microbial peptide [34].

Host genetic variations can alter both the risk for respiratory disease and severity of infection [9, 36]. Several studies have assessed the relationship between host genetic variants and TB [37, 38]. The vast majority of these studies were conducted in adults, and association between host genetic variants and risk of TB in pediatric populations remains unknown [8, 14]. A meta-analysis of 23 studies showed an increased risk for TB associated with VDR polymorphisms [12]. The Ff genotypes of *FokI* polymorphism were associated with increased risk and the bb genotype of *BsmI* genotype with decreased risk of TB. Interestingly, this was only observed in Asian populations. There does not appear to be an association between polymorphisms in CYP27B1 which encodes 25-hydroxyvitamin D3 1-alpha-hydroxylase, an enzyme catalyzing the hydroxylation of 25-hydroxyvitamin D to 1,25-dihydroxyvitamin D (the bioactive form of vitamin D)[39]. Our study extends these findings to pediatric populations in high TB and HIV burden settings. Children expressing the *TMEM16J* (rs7111432) AA-allele or the *PKP3* (rs10902158) GG-allele had a nearly 2-fold increased risk of any TB or death. However, we did not find a significant association between CYP27B1, rs7041, rs4588, *Fok1* and *Bsml* SNPs and risk of TB or death. We did however find a trend towards significance with VDBP rs7041 and probable/definite TB or death, not found when adding possible TB to the outcome. The low prevalence of the rs7041 GG/GT allele (43/346, 12.8%) may have contributed to the lack of consistency in direction of results for this SNP, but the presence of some signals in this cohort suggests the need for further study of this particular SNP in larger cohorts.

The modification of the association between host genetic variants and TB by vitamin D status has been observed in a series of studies in adults. Increased TB risk in adults was associated with the TT or Tt alleles of *TaqI* and the ff allele of *FokI*, but only in persons with vitamin D deficiency [10]. However while Gc genotypes (which are encoded in the VDBP) are not generally associated with active TB, Martinueau and colleagues observed that the Gc2/2 genotype is strongly associated with development of active TB compared with Gc1 but only in Gujurati Indians and specifically in those with low vitamin D [21]. TLRs play an important role in pathogen recognition and triggering host innate immune responses [40]. Therefore, we hypothesized that genetic variants within the *PKP3-SIGIRR-TMEM16J* gene region that affect TLR expression recently identified to be associated with susceptibility to TB would exacerbate the risk of TB in children with low vitamin D levels. Our findings support this supposition and demonstrate a strong synergy between low vitamin D levels and specific SNPs within the *PKP3-SIGIRR-TMEM16J* gene region, and the risk for TB and death among children. Young children in our study with both low vitamin D levels and rs7111432-AA or rs10902158-GG genotypes had a 3- to 8-fold increased risk of TB or death, an association much higher than that observed for low vitamin D concentrations or *PKP3-SIGIRR-TMEM16J* SNPs alone. It is therefore possible that children with SNPs in the *PKP3-SIGIRR-TMEM16J* gene region may selectively benefit from anti-mycobacterial activity of vitamin-D and therefore may potentially be prime targets for vitamin-D supplementation.

Our study has a few limitations. The majority of our children were formula fed which at the time was the standard of care in South Africa for HIV-exposed children. Breastfed children tend to have lower vitamin D concentrations than formula fed children as formula has some vitamin D supplementation. Therefore, our prevalence of low vitamin D is likely an underestimate since now the national policy has changed back to promoting exclusive breastfeeding. Our study data were from a well-conducted clinical trial where TB and death were rigorously evaluated. However, we noted that many deaths were due to infectious etiologies other than TB (diarrhea, respiratory, sepsis), and therefore our findings are consistent with the fact that the host variants associated with innate immunity were indeed relevant to both TB and other infections. Another limitation to our study is that the genetic associations identified may not be due to *PKP3* or *TMEM16J* SNPs. As noted by Horne et al.,[15] the SNPs identified as important in *PKP3* or *TMEMJ16* may be in linkage disequilibrium with SNPs within *SIGIRR*. The SIGIRR (also known as TIR8) region is a biologically important modulator of TLR-IL-1R signaling, inhibits NF-kappa B, modulates inflammation and in the mouse model has been shown to regulate harmful responses to infections such as TB [16]. The *PKP3/TMEM16J* flank the *SIGIRR* but have unknown functions. Thus it is possible that other SNPs within the *SIGIRR* region are responsible for the direct effects on microbial pathogenesis or that *PKP3/TMEM16J* regions have as yet an unidentified role in innate immunity. Finally, the study participants in this analysis were not fully representative of the parent P1041 study population due to differences in eligibility for analysis with respect to certain participant characteristics and sample availability. However, these differences are unlikely to change our overall interpretation of the associations we detected.

The World Health Organization and United Nations Millennium Development Goals call for dramatic reductions in pediatric TB cases and childhood mortality, respectively. Identifying children at high risk for TB or death therefore is a global priority. We found an association of vitamin D levels with the risk for development of TB or death in HIV-infected and –exposed but uninfected infants in South Africa. Additionally, specific variants within the *PKP3-SIGIRR-TMEM16J* gene region were also associated with risk of TB or death. More importantly, genetic variants combined with low vitamin D status identified infants at highest risk for TB disease or death. These findings suggest that vitamin D supplementation of infants may be of benefit to infants in high TB burden countries, and that children with specific genetic variations may benefit from supplementation to a greater extent. Clinical trials are needed to evaluate the benefits of supplemental vitamin D in high-risk settings.

## Supporting Information

S1 Table Comparison between P1041 participants with plasma/PBMC samples (eligible for subcohort) and those without. (DOCX)

S2 Table Genetic polymorphisms by vitamin D status among subcohort. (DOCX)

S3 Table Adjusted hazard ratios of other genetic polymorphisms as potential risk factors for study outcomes. Models adjust for HIV status, sex, mother TB diagnosis history, type of housing, breastfeeding (excluded in model for probable or definite TB), weight z-score, site, and randomized treatment group (INH or placebo) *CI: Confidence interval.
(DOCX)
