# Supplementary material for: Low Vitamin-D Levels Combined with PKP3-SIGIRR-TMEM16J Host Variants Is Associated with Tuberculosis and Death in HIV-Infected and -Exposed Infants
Source: PLoS One. 2016 Feb 12;11(2):e0148649. doi: 10.1371/journal.pone.0148649 (PMC4752266; doi:10.1371/journal.pone.0148649)
Supplement: S1 Table — (DOCX) [file pone.0148649.s001.docx]

**S1 Table:** Comparison between P1041 participants with plasma/PBMC samples (eligible for subcohort) and those without.

|  | | | **Had plasma and PBMC for consideration of entry into subcohort** | |  |
| --- | --- | --- | --- | --- | --- |
| **Characteristic** |  | **Total (N=1351)** | **No (N=534)** | **Yes (N=817)** | **P-Value** |
| Year of randomization | 2004 | 5 (0%) | 5 (1%) | 0 (0%) | <0.001* |
|  | 2005 | 715 (53%) | 337 (63%) | 378 (46%) |  |
|  | 2006 | 247 (18%) | 62 (12%) | 185 (23%) |  |
|  | 2007 | 252 (19%) | 63 (12%) | 189 (23%) |  |
|  | 2008 | 132 (10%) | 67 (13%) | 65 (8%) |  |
|  | | | | | |
| HIV status | HIV-infected | 543 (40%) | 174 (33%) | 369 (45%) | <0.001* |
|  | HIV-uninfected | 808 (60%) | 360 (67%) | 448 (55%) |  |
|  | | | | | |
| Site | Johannesburg | 878 (65%) | 203 (38%) | 675 (83%) | <0.001* |
|  | Cape Town | 409 (30%) | 324 (61%) | 85 (10%) |  |
|  | 12701 | 4 (0%) | 4 (1%) | 0 (0%) |  |
|  | Durban | 60 (4%) | 3 (1%) | 57 (7%) |  |
|  | | | | | |
| Sex | Male | 648 (48%) | 254 (48%) | 394 (48%) | 0.81* |
|  | Female | 703 (52%) | 280 (52%) | 423 (52%) |  |
|  | | | | | |
| Weight z-score (WHO) | Mean (s.d.) | -0.67 (1.40) | -0.48 (1.39) | -0.79 (1.39) | <0.001** |
|  | Median (Q1, Q3) | -0.56 (-1.45, 0.28) | -0.42 (-1.30, 0.48) | -0.67 (-1.57, 0.14) |  |
|  | | | | | |
| Birth Weight<2500 gms | Yes | 222 (16%) | 89 (17%) | 133 (16%) | 0.85* |
|  | No | 1,129 (84%) | 445 (83%) | 684 (84%) |  |
|  | | | | | |
| Mother Ever had TB Dx | Yes | 97 (7%) | 37 (7%) | 60 (7%) | 0.77* |
|  | No | 1,254 (93%) | 497 (93%) | 757 (93%) |  |
|  | | | | | |
| Housing Type | Formal (brick) house | 813 (60%) | 269 (50%) | 544 (67%) | <0.001* |
|  | Informal (shack/wooden) | 534 (40%) | 263 (49%) | 271 (33%) |  |
|  | | | | | |
| Water access | Tap(running water) inside house | 447 (33%) | 174 (33%) | 273 (33%) | <0.001* |
|  | Tap(running water) on plot, single use | 555 (41%) | 165 (31%) | 390 (48%) |  |
|  | Communal tap, multiple households | 349 (26%) | 195 (37%) | 154 (19%) |  |
|  | | | | | |
| Study participant ever breastfed? | Yes | 121 (9%) | 39 (7%) | 82 (10%) | 0.09* |
|  | No | 1,230 (91%) | 495 (93%) | 735 (90%) |  |
|  | | | | | |
| ***Chi-Square Test **Wilcoxon Test** | | | | | |
