# Supplementary material for: Low Vitamin-D Levels Combined with PKP3-SIGIRR-TMEM16J Host Variants Is Associated with Tuberculosis and Death in HIV-Infected and -Exposed Infants
Source: PLoS One. 2016 Feb 12;11(2):e0148649. doi: 10.1371/journal.pone.0148649 (PMC4752266; doi:10.1371/journal.pone.0148649)
Supplement: S2 Table — (DOCX) [file pone.0148649.s002.docx]

**S2 Table:** Genetic polymorphisms by vitamin D status among subcohort.

|  | | | **Insufficient Vitamin D (<32 ng/mL)** | |  |
| --- | --- | --- | --- | --- | --- |
| **Characteristic** |  | **Total (N=270)** | **No (N=200)** | **Yes (N=70)** | **P-Value*** |
|  | | | | | |
| ANO9 | AA | 69 | 54 (78%) | 15 (22%) | 0.31 |
|  | AG/GG | 196 | 141 (72%) | 55 (28%) |  |
|  | Missing | 5 | 5 (.%) | 0 (.%) |  |
|  | | | | | |
| CYP2R1 | AA/AG | 90 | 64 (71%) | 26 (29%) | 0.51 |
|  | GG | 175 | 131 (75%) | 44 (25%) |  |
|  | Missing | 5 | 5 (.%) | 0 (.%) |  |
|  | | | | | |
| DHCR7 | GG | 179 | 127 (71%) | 52 (29%) | 0.16 |
|  | GT/TT | 86 | 68 (79%) | 18 (21%) |  |
|  | Missing | 5 | 5 (.%) | 0 (.%) |  |
|  | | | | | |
| PKP3 (A/G) | AA/AG | 150 | 108 (72%) | 42 (28%) | 0.50 |
|  | GG | 115 | 87 (76%) | 28 (24%) |  |
|  | Missing | 5 | 5 (.%) | 0 (.%) |  |
|  | | | | | |
| PKP3 (C/T) | CC/CT | 140 | 104 (74%) | 36 (26%) | 0.78 |
|  | TT | 125 | 91 (73%) | 34 (27%) |  |
|  | Missing | 5 | 5 (.%) | 0 (.%) |  |
|  | | | | | |
| VDBP | AA | 232 | 172 (74%) | 60 (26%) | 0.59 |
|  | AC/CC | 33 | 23 (70%) | 10 (30%) |  |
|  | Missing | 5 | 5 (.%) | 0 (.%) |  |
|  | | | | | |
| VDR bsm | AA/AG | 108 | 82 (76%) | 26 (24%) | 0.47 |
|  | GG | 157 | 113 (72%) | 44 (28%) |  |
|  | Missing | 5 | 5 (.%) | 0 (.%) |  |
|  | | | | | |
| VDR fok-1 | CC | 186 | 138 (74%) | 48 (26%) | 0.73 |
|  | CT/TT | 79 | 57 (72%) | 22 (28%) |  |
|  | Missing | 5 | 5 (.%) | 0 (.%) |  |
|  | | | | | |
| rs4588 | AC | 29 | 19 (66%) | 10 (34%) | 0.30 |
|  | CC | 236 | 176 (75%) | 60 (25%) |  |
|  | Missing | 5 | 5 (.%) | 0 (.%) |  |
|  | | | | | |
| rs7041 | GG/GT | 34 | 23 (68%) | 11 (32%) | 0.40 |
|  | TT | 231 | 172 (74%) | 59 (26%) |  |
|  | Missing | 5 | 5 (.%) | 0 (.%) |  |
|  | | | | | |
| ***Chi-Square Test** | | | | | |
