# Supplementary material for: Low Vitamin-D Levels Combined with PKP3-SIGIRR-TMEM16J Host Variants Is Associated with Tuberculosis and Death in HIV-Infected and -Exposed Infants
Source: PLoS One. 2016 Feb 12;11(2):e0148649. doi: 10.1371/journal.pone.0148649 (PMC4752266; doi:10.1371/journal.pone.0148649)
Supplement: S3 Table — Models adjust for HIV status, sex, mother TB diagnosis history, type of housing, breastfeeding (excluded in model for probable or definite TB), weight z-score, site, and randomized treatment group (INH or placebo) *CI: Confidence interval. (DOCX) [file pone.0148649.s003.docx]

**S3 Table:** Adjusted hazard ratios of other genetic polymorphisms as potential risk factors for study outcomes.

| **Outcome** | **Genotype** | **Variant** | **Hazards Ratio (95% CI*)** | | **P-value** |  |
| --- | --- | --- | --- | --- | --- | --- |
| Probable/definite TB |  |  |  | |  |  |
|  | CYP2R1 | GG (vs. AA/AG) | 1.00 (0.49,2.05) | | 0.99 |  |
|  | DHCR7 | GT/TT (vs. GG) | 1.00 (0.50,1.98) | | 1.00 |  |
|  | VDBP | AC/CC (vs. AA) | 0.96 (0.37,2.54) | | 0.94 |  |
|  | VDR bsm | GG (vs. AA/AG) | 0.95 (0.48,1.87) | | 0.88 |  |
|  | VDR fok-1 | CT/CC (vs. CC) | 1.04 (0.50,2.16) | | 0.92 |  |
|  | rs4588 | CC (vs AC) | 1.02 (0.36,2.84) | | 0.97 |  |
|  | rs7041 | TT (vs. GG/GT) | 3.28 (0.77,14.0) | | 0.11 |  |
| Probable/definite TB or death | |  | |  |  | |
|  | CYP2R1 | GG (vs. AA/AG) | 1.01 (0.53,1.92) | | 0.98 |  |
|  | DHCR7 | GT/TT (vs. GG) | 1.05 (0.52,2.11) | | 0.90 |  |
|  | VDBP | AC/CC (vs. AA) | 0.94 (0.38,2.32) | | 0.90 |  |
|  | VDR bsm | GG (vs. AA/AG) | 1.12 (0.59,2.14) | | 0.73 |  |
|  | VDR fok-1 | CT/CC (vs. CC) | 0.98 (0.50,1.93) | | 0.96 |  |
|  | rs4588 | CC (vs AC) | 0.98 (0.40,2.39) | | 0.97 |  |
|  | rs7041 | TT (vs. GG/GT) | 3.25 (0.95,11.1) | | 0.06 |  |
| Any TB |  |  |  | |  |  |
|  | CYP2R1 | GG (vs. AA/AG) | 0.90 (0.58,1.40) | | 0.65 |  |
|  | DHCR7 | GT/TT (vs. GG) | 0.95 (0.62,1.47) | | 0.83 |  |
|  | VDBP | AC/CC (vs. AA) | 0.74 (0.38,1.44) | | 0.38 |  |
|  | VDR bsm | GG (vs. AA/AG) | 0.98 (0.65,1.48) | | 0.93 |  |
|  | VDR fok-1 | CT/CC (vs. CC) | 0.86 (0.55,1.35) | | 0.52 |  |
|  | rs4588 | CC (vs AC) | 1.04 (0.55,1.97) | | 0.90 |  |
|  | rs7041 | TT (vs. GG/GT) | 1.05 (0.57,1.94) | | 0.87 |  |
| Any TB or death |  |  |  | |  |  |
|  | CYP2R1 | GG (vs. AA/AG) | 0.92 (0.60,1.40) | | 0.70 |  |
|  | DHCR7 | GT/TT (vs. GG) | 0.95 (0.62,1.46) | | 0.81 |  |
|  | VDBP | AC/CC (vs. AA) | 0.77 (0.40,1.46) | | 0.42 |  |
|  | VDR bsm | GG (vs. AA/AG) | 1.02 (0.68,1.53) | | 0.92 |  |
|  | VDR fok-1 | CT/CC (vs. CC) | 0.86 (0.56,1.33) | | 0.49 |  |
|  | rs4588 | CC (vs AC) | 0.99 (0.54,1.83) | | 0.98 |  |
|  | rs7041 | TT (vs. GG/GT) | 1.14 (0.62,2.10) | | 0.67 |  |

*Models adjust for HIV status, sex, mother TB diagnosis history, type of housing, breastfeeding (excluded in model for probable or definite TB), weight z-score, site, and randomized treatment group (INH or placebo) *CI: Confidence interval*
